# Digital Technologies in a Post-Pandemic Southeast Asia: Measures for Enhancing Regional Approaches

**DOI:** 10.12688/f1000research.165762.1

**Published:** 2025-06-25

**Authors:** Bama Andika Putra

**Affiliations:** 1 University of Bristol School of Sociology Politics and International Studies, Bristol, England, UK; 2 International Relations, Universitas Hasanuddin Fakultas Ilmu Sosial dan Ilmu Politik, Makassar, South Sulawesi, Indonesia

**Keywords:** Digital Technologies, Telehealth, Policy Analysis, Southeast Asia, ASEAN, Regional Approaches

## Abstract

The Association of Southeast Asian Nations (ASEAN) has taken some measures to advance collective action to accelerate telehealth in the region. Still, it has encountered the problem of digital readiness and digital health preparedness disparities within the region. To maintain the consolidation of digital health utilization among ASEAN member states, this article offers policy recommendations that help to address the diverse approaches taken and compensate for the capacity diversities among the members. Drawing on insights from published official policies, government health ministry websites of Southeast Asian nations, and ASEAN’s digital health policies, the article first reviews the diversities of digital technologies in a post-pandemic Southeast Asia and then assess measures to enhance regional approaches. Several recommendations are presented: First, ASEAN can standardize regional frameworks on telehealth through the sharing of digital health transformation blueprints and the utilization of ASEAN and ASEAN Plus forums to bridge different understandings and advance the region’s digital health initiatives. Second, ASEAN facilitates investments through a telehealth sandbox and fosters collaborations among different stakeholders. Although the recommendations are consistent with the ‘ASEAN Way,’ concerns over different commitments and expectations, risks of privacy infringements, and misuse of technology in the authoritarian states of the region will be lingering concerns to Southeast Asia’s telehealth landscape.

## 1. Introduction

Southeast Asia was among the region’s severely affected by the COVID-19. The first cases in the region were reported in January 2020, ^1^ leading states to undertake lockdown and travel restrictions to curtail the spread of the SARS-CoV-2 virus. The ASEAN reported that a staggering 37,003,383 people in the region were positive for COVID-19, with ASEAN experiencing a sharp economic contraction. ^2, 3^ Nevertheless, after the pandemic’s peak, things started to calm down, and ASEAN recorded a commendable 4.1 percent growth in 2023, which was above the global average. ^2^ ASEAN member states were committed to rebuilding their economies and were devoted to adopting the ASEAN Comprehensive Recovery Framework (ACRF) to recover. Among the provisions within the ACRF has been the advancement of digitalization to reinforce the region’s public health systems.

Indeed, there is a clear nexus of technology and healthcare. Often termed ‘telehealth,’ ASEAN member states have utilized this to provide cost-effective health care for those in isolated communities, a prominent feature within the Southeast Asian demography. In the past years, telehealth (41%) and predictive analytics (43%) have generated the most capital investments within the ASEAN health technology landscape. ^4^ Vis-à-vis the COVID-19, telehealth had a prominent role in managing the outbreaks. ASEAN member states adopting high surveillance and contact tracing had the lowest rates of COVID-19 mortality. ^5, 6^ There have also been favorable perceptions of telehealth, as the internet of things, artificial intelligence, big data, and blockchain have immense positive impacts in establishing a cohesive public health strategy due to its role in the prevention, detection, and surveillance of the virus. ^1, 7, 8^

However, Southeast Asia is a profoundly diverse region. A look into CISCO’s Digital Readiness Index in 2021 ( Table 1 below) shows how the regions’ nations have diverse standards and potential in leveraging telehealth in the future by considering human capital, technology adoption, technology infrastructure, and other influential elements that could affect the telehealth landscape of a country. The results contrast among member states, with Singapore attaining a first-world rank, while Myanmar and Laos attaining scores recorded as some of the lowest globally. ^9^

**Table 1.** Digital readiness index 2021.

ASEAN member state	World rank	Score	DGI index category
Brunei	NA	NA	NA
Cambodia	92	-0.38	Accelerate (low)
Indonesia	73	-0.06	Accelerate (low)
Laos	115	-0.89	Accelerate (low)
Malaysia	42	+0.46	Accelerate (high)
Myanmar	110	-0.85	Accelerate (low)
Philippines	87	-0.25	Accelerate (low)
Thailand	51	+0.32	Accelerate (high)
Singapore	1	+2.37	Amplify
Vietnam	57	+0.22	Accelerate (high)

Source: Adopted from the CISCO’s Digital Readiness Index (2021).

A contributor to such disparities is the ASEAN member states’ undertaking of telehealth initiatives at their own pace and initiative. Take, for example, health consultation applications such as Halodoc (Indonesia), elderly health monitoring such as DoCare Protect (Thailand), coordination of community support for dementia patients like Dementia Friends application (Singapore), and the diagnosing of patients through chat such as OneNUHS Health Chatbox (Singapore) and CekMata (Indonesia). ^10– 14^ During the COVID-19 pandemic, Indonesia’s PeduliLindungi and Malaysia’s Mysejahtera were influential in providing individual COVID-19-related information to all its citizens. ^15^ As Clavier and Ghesquiere mentioned, “Digital solutions have been the signature of Southeast Asia’s response to COVID-19”. ^1^

This article argues that maintaining the consolidation of digital health utilization among ASEAN member states needs to address the challenges of diverse approaches and capacity diversities. After COVID-19, the rush to make innovations in telehealth is not likely to slow down. The question then lies in how the regional organization of ASEAN can further facilitate the dynamic sandbox of health technology-related innovations to excel in digital solutions to the region’s health landscape. It asks what ASEAN can do in a post-pandemic Southeast Asian setting.

## 2. ASEAN: The challenge of disparity in telehealth

Digital solutions have been a prominent feature in Southeast Asia’s health landscape. Collectively, ASEAN and ASEAN member states learned from COVID-19 and established the ASEAN BioDiaspora Virtual Center that would enhance the regional organization’s response and preparedness for epidemics and pandemics in the future. ^15^ As slightly shown in the previous section, however, on an individual scale, Southeast Asian states have taken different approaches to their telehealth. They fulfill the functions of communication, aid distribution, digital contract tracing, and self-reporting of symptoms concerning pandemics and health concerns. ^1^ The current state of the ASEAN member states can be seen in the Global Development Incubator’s Global Digital Health Monitor, 2022 ( Table 2 below). Southeast Asia’s digital health landscape is so diverse that some states are found in Phase 1 (no coordinating body exists on digital health care/nascent governance structure) and some in Phase 5 (digital health and data governance institutionalized). ^16^

**Table 2.** Global digital health monitor 2022.

ASEAN member state	Legislation, policy and compliance	Infrastructure	Leadership and governance	Workforce	Services and applications	Strategy and investment	Standards and operability	GDHM overall score
Brunei	Phase 3	Phase 4	NA	NA	NA	NA	NA	Phase 4
Cambodia	Phase 2	Phase 2	Phase 2	Phase 1	Phase 2	Phase 1	Phase 2	Phase 2
Indonesia	Phase 4	Phase 4	Phase 4	Phase 3	Phase 4	Phase 3	Phase 3	Phase 4
Laos	Phase 3	Phase 4	Phase 4	Phase 2	Phase 3	Phase 3	Phase 3	Phase 3
Malaysia	Phase 5	Phase 4	Phase 5	Phase 4	Phase 4	Phase 4	Phase 3	Phase 4
Myanmar	Phase 1	Phase 2	Phase 3	Phase 1	Phase 3	Phase 4	Phase 2	Phase 2
Philippines	Phase 4	Phase 4	Phase 2	Phase 2	Phase 4	Phase 4	Phase 4	Phase 4
Thailand	Phase 5	Phase 3	Phase 5	Phase 4	Phase 5	Phase 4	Phase 3	Phase 4
Singapore	Phase 5	Phase 5	Phase 5	NA	NA	NA	NA	Phase 5
Vietnam	NA	NA	NA	NA	NA	NA	NA	NA

Source: Adopted from the Global Development Incubator (2022).

Digital health Initiatives are a complex topic. It needs to consider different variables such as infrastructure, ICT, regulation, and digital literacy, which Table 1 shows is severely different among the ASEAN member states. As ASEAN reported in 2024, “Currently, there is no uniformity in the current landscape of digital health readiness among the ASEAN member states”. ^15^ Regarding leadership and governance, ASEAN is encountering underinvestment in its private telehealth sectors and slow adjustment in government policies. ^17^ In advancing the telehealth landscape strategy, only half of the ASEAN member states have a national roadmap elaborating on the nation’s digital health transformations. ^7^ Legislation that caters to the development of digital health also remains a vital barrier to the telehealth transformation of Southeast Asia, with some questioning uneven data protection regulation and general lack of policies. ^11, 18^ Minus Singapore, ASEAN member states still lack sufficient ICT infrastructure, such as internet speed. ^9, 16^

Digitalization efforts are not distributed evenly in Southeast Asia. The lack of capacity disparity and technological readiness severely impacts the capacity to respond to similar public health crises in the future, such as the magnitude of COVID-19. The region hosts high technological maturity states that are digital pioneers in telehealth, such as Singapore, but simultaneously host countries with some of the lowest digital literacy and penetration, such as Cambodia, Myanmar, and Lao PDR. The following section will explore some recommendations that ASEAN could undertake as a regional grouping and provide the suggested actionable recommendations.

## 3. The way forward: Policy recommendations and the moderate-level solution proposed

Despite the disparities identified in this article, there is room to narrow the gaps and provide an ideal telehealth landscape for the Southeast Asian region through ASEAN. The first recommendation offered is standardizing the regulatory framework. This policy considers that the vast differences existing in national laws that lead ASEAN to be unable to adopt a transnational telehealth scheme. It is therefore recommended that ASEAN member states share digital health transformation blueprints among members, which can be coordinated first in ASEAN forums that involve the Ministry of Health of ASEAN member states and ASEAN Plus forums (‘ASEAN Plus Three’ for example, would include China, South Korea, and Japan). By synergizing efforts, countries can establish long-term plans to develop relevant government institutions and telehealth programs that would advance the readiness of ASEAN member states in post-pandemic times.

A standardized regulatory framework would also establish equal grounds for cybersecurity measures. Since telehealth is a substantial technological element in its development, the threat of cybersecurity is inevitable. This first recommendation should cater towards such threat-possible scenarios by ASEAN fostering cybersecurity frameworks such as a single certification approach for the Southeast Asian region. Doing so ensures that both ASEAN member states and the private sectors can build synergy in their programs, as slightly mentioned in the ASEAN Digital Masterplan 2025. ^15^

The second recommendation is for ASEAN to enhance investments in the digital health sector. Considering that most digital health initiatives during COVID-19 originated from the private sector, it would be ideal for ASEAN to establish a telehealth sandbox that is financially supported by private sectors working on specific telehealth elements in the region. Collaborating with ASEAN member state governments, donors, and private sectors allows more significant exposure to the importance of digital health initiatives for Southeast Asia, allowing the exploration of new technological innovations. Since Indonesia’s ASEAN chairmanship in 2023, ASEAN has placed great emphasis on the ASEAN Indo-Pacific Forum (AIPF) as a platform for government and private sectors to attract foreign investments from ASEAN (and partnering states). ^19– 21^ Platforms such as the AIPF can be fully utilized to address the lack of readily available finances to explore telehealth opportunities within the region.

Investments in the digital health sector should also consider the issues of digital disparity among the ASEAN member states. There is, therefore, the need to address more grassroots concerns on Southeast Asian people’s digital readiness through the provision of high-speed accessible internet, digital health literacy training and education, knowledge transfers, and capacity-building measures on user-friendly applications.

Recommendations one and two offer a number of positive benefits for ASEAN and its member states. Most importantly, the recommendation to standardize the regulatory framework and facilitate investments in the digital health sector is consistent with the ‘ASEAN Way.’ The ASEAN Way is ASEAN’s foundational norms in the conduct of international relations in the Southeast Asian region, by placing heavy emphasis on the importance of non-interference, consensus decision-making, and pacific settlement of disputes. ^22– 24^ Telehealth is a sector that has shown to be vital for the Southeast Asian region and will continue to be in a post-pandemic health landscape. The recommended provisions ensure that it does not impose policies in which ASEAN member states would be pressured to undertake pre-determined policies concerning the digital health sector. It merely continues existing efforts to explore other means to accelerate the telehealth landscape in Southeast Asia through ASEAN.

Nevertheless, the proposed recommendations could have some adverse outcomes. For the first recommendation, there should be an expectation of differing commitments displayed in the need to regulate the digital health initiative in Southeast Asia. Some ASEAN member states have a strong prospect of emphasizing traditional interventions in medical care ^25^; meanwhile, others may not have the financial capacity to commit to a single framework. The regional organization’s development gap and disparity among ASEAN members are recurring themes. ^26– 28^ The term ‘CMLV’ is used to represent the fragility of the newer members of ASEAN, Cambodia, Myanmar, Laos, and Vietnam, abiding by ASEAN’s fast-paced norm development. ^27^ A look into the digital readiness and digital health landscape ( Tables 1 and 2) also shows that the CMLV is among the states that struggle to display readiness to advance its digital health sectors. ^9, 16^ The disparity of digital readiness within a state’s borders should also be considered. As Das and Zhang mentioned at the start of the COVID-19 pandemic, those not digitally versed (within a state) have struggled with the vast digital responses imposed by their governments. ^29^

Another concern that equally affects recommendations one and two is the implications of the increased surveillance tools in telehealth. A critical feature of the Southeast Asian region is the diversity of government systems among the member states, leading to diverse human rights appreciation. If investments are directed to programs that advance application surveillance tools in digital check-in systems and contact tracing technologies, there would be a concern about privacy infringements. ASEAN is known to be inconsistent with its human rights standards, seeing the heavy emphasis placed on the promotion of human rights (rather than protection) in the ASEAN Intergovernmental Commission on Human Rights, criticisms over the culture and sovereignty-subjected human rights outlined in the ASEAN Human Rights Declaration, and the blind-eye placed in human rights atrocities taking place in Myanmar. ^30– 33^ The second recommendation aims to accelerate the telehealth landscape of Southeast Asian states. Considering the sensitivity of human rights in the more authoritarian states of ASEAN, the intention to advance digital health could severely backlash the organization if such technologies are not balanced with proper care over privacy rights.

Because of the positive and negative implications of the two recommendations, this article suggests undertaking moderate changes to the Southeast Asian telehealth landscape. The impact of digital initiatives on maintaining order and assisting policymakers in responding to pandemics has been undeniable. Nevertheless, Southeast Asia is a unique region. First, Southeast Asia’s demography shows a significant disparity in digital readiness (both government and society). Second, the sensitivity of Southeast Asia to human rights discourses demonstrates the importance of not overly imposing policies that can potentially undermine human rights conditions within a state’s borders. The best measure, therefore, is to moderately adopt the first recommendation by ASEAN standardizing regulatory framework at a pace acceptable to all members. Second, investments in the telehealth sector should ensure that the policies have explicit provisions to counter the possibility of the misuse of technology (especially by ASEAN member states with authoritarian settings).

## 4. Conclusion

The digital technology landscape of Southeast Asia is rapidly growing. Lessons from the COVID-19 pandemic showed that Southeast Asia is among the regions that heavily emphasize the nexus between technologies and health care, aiming to efficiently provide better nationwide health services. Nevertheless, a significant disparity was identified among the Southeast Asian states regarding digital readiness and the pace of digital health initiatives shown among the states. As Southeast Asia’s leading regional group, ASEAN has the potential to address some lingering concerns that have impeded the region from accelerating its telehealth to increased heights.

This article recommends measures that ASEAN could adopt. First, the regional framework of ASEAN’s telehealth is standardized by sharing digital health transformation blueprints and utilizing ASEAN and ASEAN Plus forums to bridge different understandings and advance the region’s digital health initiatives. Second is ASEAN to facilitate investments in the digital health sector, primarily through a telehealth sandbox and facilitating finance collaborations between different sectors. Nevertheless, it is also pivotal to balance the various pros and cons of the policies. Although both recommendations are consistent with the ASEAN Way, there are still lingering concerns over different commitment expectations among ASEAN member states, differing capacities of the CMLV, and the risks of privacy infringements and misuse of technology by authoritarian states in the region. It is, therefore, recommended that a moderate pace be adopted in Southeast Asia’s telehealth development. Despite the positive prospects, ensuring the recommendations are in tune with Southeast Asia’s unique political landscape should be ASEAN’s top priority.

## Ethical approval

Ethical approval is no required for this article.

## Data Availability

The dataset used in this study is publicly available and sourced from reputable organizations. All data can be accessed through their official platforms, with the detail data and links accessible below:
• CISCO Digital Readiness Index: https://www.cisco.com/c/m/en_us/about/corporate-social-responsibility/research-resources/digital-readiness-index.html#/; • Global Digital Health Monitor: https://digitalhealthmonitor.org/stateofdigitalhealth23 All data required to replicate the findings of this study are available in those websites, in which users can filter based on the inquired variable and period.
