# Digital Technologies in a Post-Pandemic Southeast Asia: Measures for Enhancing Regional Approaches

**DOI:** 10.12688/f1000research.165762.2

**Published:** 2025-12-01

**Authors:** Bama Andika Putra

**Affiliations:** 1 University of Bristol School of Sociology Politics and International Studies, Bristol, England, UK; 2 International Relations, Universitas Hasanuddin Fakultas Ilmu Sosial dan Ilmu Politik, Makassar, South Sulawesi, Indonesia

**Keywords:** Digital Technologies, Telehealth, Policy Analysis, Southeast Asia, ASEAN, Regional Approaches

## Abstract

The Association of Southeast Asian Nations (ASEAN) has taken some measures to advance collective action to accelerate telehealth in the region. Still, it has encountered the problem of digital readiness and digital health preparedness disparities within the region. To maintain the consolidation of digital health utilization across ASEAN member states, this article offers policy recommendations to address the diverse approaches taken and compensate for capacity differences among members. Drawing on insights from published official policies, government health ministry websites of Southeast Asian nations, and ASEAN’s digital health policies, the article first reviews the diversities of digital technologies in a post-pandemic Southeast Asia and then assesses measures to enhance regional approaches. Several recommendations are presented: First, ASEAN can standardize regional frameworks for telehealth by sharing digital health transformation blueprints and leveraging ASEAN and ASEAN Plus forums to bridge divergent understandings and advance the region’s digital health initiatives. Second, ASEAN facilitates investment through a telehealth sandbox and fosters collaboration among stakeholders. Although the recommendations are consistent with the ‘ASEAN Way,’ lingering concerns in Southeast Asia’s telehealth landscape include different commitments and expectations, risks of privacy infringements, and the misuse of technology in the region’s authoritarian states.

## 1. Introduction

Southeast Asia was among the regions severely affected by COVID-19. The first cases in the region were reported in January 2020, leading states to implement lockdowns and travel restrictions to curb the spread of the SARS-CoV-2 virus. The ASEAN reported that a staggering 37,003,383 people in the region were positive for COVID-19, and that the region experienced a sharp economic contraction. ^1, 2^ Nevertheless, after the pandemic’s peak, things started to calm down, and ASEAN recorded a commendable 4.1 percent growth in 2023, which was above the global average. ^1^ ASEAN member states were committed to rebuilding their economies and were devoted to adopting the ASEAN Comprehensive Recovery Framework (ACRF) to recover. Among the provisions within the ACRF has been the advancement of digitalization to reinforce the region’s public health systems.

Indeed, there is a clear nexus of technology and healthcare. Often termed ‘telehealth,’ ASEAN member states have used it to provide cost-effective health care to people in isolated communities, a prominent feature of Southeast Asian demography. In recent years, telehealth (41%) and predictive analytics (43%) have generated the most capital investments in the ASEAN health technology landscape. ^3^ Vis-à-vis COVID-19, telehealth had a prominent role in managing the outbreaks. ASEAN member states that adopted high surveillance and contact tracing had the lowest COVID-19 mortality rates. ^4, 5^ There have also been favorable perceptions of telehealth, as the internet of things, artificial intelligence, big data, and blockchain have immense positive impacts on establishing a cohesive public health strategy, due to their roles in the prevention, detection, and surveillance of the virus. ^6– 8^

However, Southeast Asia is a profoundly diverse region. A look into the International Telecommunication Union’s (ITU) 2024 Information, Technology, and Communication (ICT) Development Index ( Table 1 below) reveals how the region’s nations have diverse conditions based on the pillars of universal connectivity (indicators related to the people, households, communities, and business) and indicators of the enablers of connectivity (infrastructure, affordability, safety and security, device, and skills). ^10^ The comparison below compares the scores between 2023 and 2024 to provide an overview of changes and trends in the region.

**Table 1.** Comparison of the ICT Development Index Score of ASEAN member states (2023, 2024).

ASEAN Member State	ICT Development Index Score 2023	ICT Development Index Score 2024
Brunei Darussalam	94.8	95.7
Cambodia	68.5	72.6
Indonesia	80.1	82.8
Laos	64.6	65.3
Malaysia	94.5	95.0
Myanmar	65.7	63.8
Philippines	65.0	74.4
Thailand	88.7	91.0
Singapore	97.4	97.8
Vietnam	80.6	85.0

Source: Adopted from the ITU ICT Development Index. ^9^

A contributor to such disparities is the ASEAN member states’ undertaking of telehealth initiatives at their own pace and initiative. Take, for example, health consultation applications such as Halodoc (Indonesia), elderly health monitoring such as DoCare Protect (Thailand), coordination of community support for dementia patients like Dementia Friends application (Singapore), and the diagnosis of patients through chat such as OneNUHS Health Chatbox (Singapore) and CekMata (Indonesia). ^11– 15^ During the COVID-19 pandemic, Indonesia’s PeduliLindungi and Malaysia’s MySejahtera were influential in providing individual COVID-19-related information to all their citizens. ^16^ As Clavier and Ghesquiere mentioned, “Digital solutions have been the signature of Southeast Asia’s response to COVID-19”. ^6^ These initiatives are extensions of already successful telehealth platforms in Asia, such as India’s *eSanjeevani*, which promotes inclusivity by bridging the urban-rural divide and ensuring equitable healthcare access for all. ^16^

This article argues that maintaining the consolidation of digital health utilization among ASEAN member states requires addressing the challenges posed by diverse approaches and varying capacities. After COVID-19, the rush to develop innovations in telehealth is unlikely to slow. The question then lies in how the regional organization ASEAN can further facilitate a dynamic sandbox for health technology-related innovations to excel in digital solutions for the region’s health landscape. It asks what ASEAN can do in a post-pandemic Southeast Asian setting.

### 2. ASEAN: The challenge of disparity in telehealth

Digital solutions have been a prominent feature in Southeast Asia’s health landscape. Collectively, ASEAN and its member states learned from COVID-19 and established the ASEAN BioDiaspora Virtual Center to enhance the regional organization’s response and preparedness for future epidemics and pandemics. ^15^ As slightly shown in the previous section, however, on an individual scale, Southeast Asian states have taken different approaches to their telehealth. They fulfill the functions of communication, aid distribution, digital contract tracing, and symptom self-reporting related to pandemics and other health concerns. ^6^ The current state of the ASEAN member states can be seen in the Global Development Incubator’s Global Digital Health Monitor, 2023 ( Table 2 below). Southeast Asia’s digital health landscape is so diverse that some states are in Phase 1 (no coordinating body for digital health care/nascent governance structure) and others in Phase 5 (digital health and data governance institutionalized). ^17^

**Table 2.** Global Digital Health Monitor 2023.

ASEAN Member State	Legislation, Policy, and Compliance	Infrastructure	Leadership and Governance	Workforce	Services and Applications	Strategy and Investment	Standards and operability	GDHM Overall Score
Brunei	Phase 3	Phase 4	NA	NA	NA	NA	NA	Phase 4
Cambodia	Phase 2	Phase 2	Phase 2	Phase 1	Phase 2	Phase 1	Phase 2	Phase 2
Indonesia	Phase 4	Phase 4	Phase 4	Phase 3	Phase 4	Phase 3	Phase 3	Phase 4
Laos	Phase 3	Phase 4	Phase 4	Phase 2	Phase 3	Phase 3	Phase 3	Phase 3
Malaysia	Phase 5	Phase 4	Phase 5	Phase 4	Phase 4	Phase 4	Phase 3	Phase 4
Myanmar	Phase 1	Phase 2	Phase 3	Phase 1	Phase 3	Phase 4	Phase 2	Phase 2
Philippines	Phase 4	Phase 4	Phase 2	Phase 2	Phase 4	Phase 4	Phase 4	Phase 4
Thailand	Phase 5	Phase 3	Phase 5	Phase 4	Phase 5	Phase 4	Phase 3	Phase 4
Singapore	Phase 5	Phase 5	Phase 5	NA	NA	NA	NA	Phase 5
Vietnam	NA	NA	NA	NA	NA	NA	NA	NA

Source: Adopted from the Global Development Incubator. ^17^

Digital health Initiatives are a complex topic. It needs to consider variables such as infrastructure, ICT, regulation, and digital literacy, as Table 1 shows, which are significantly different across ASEAN member states. As ASEAN reported in 2024, “Currently, there is no uniformity in the current landscape of digital health readiness among the ASEAN member states”. ^15^ Regarding leadership and governance, ASEAN is encountering underinvestment in its private telehealth sectors and slow adjustment in government policies. ^17^ In advancing the telehealth landscape strategy, only half of the ASEAN member states have a national roadmap elaborating on the nation’s digital health transformations. ^7^ Legislation that caters to the development of digital health also remains a vital barrier to the telehealth transformation of Southeast Asia, with some questioning uneven data protection regulation and the general lack of policies. ^11, 18^ Minus Singapore, ASEAN member states still lack sufficient ICT infrastructure, including internet speeds. ^9, 16^

Digitalization efforts are not evenly distributed across Southeast Asia. The lack of capacity disparity and technological readiness severely limits the ability to respond to future public health crises of a similar magnitude to COVID-19. The region hosts high-tech states that are digital pioneers in telehealth, such as Singapore, but also countries with some of the lowest digital literacy and penetration, such as Cambodia, Myanmar, and Lao PDR. In general, issues of digital readiness related to data governance, funding inequities, and lack of ICT infrastructure remain the lingering challenges for Southeast Asian states. The following section will explore some recommendations that ASEAN could undertake as a regional grouping and provide the suggested actionable recommendations.

### 3. The way forward: Policy recommendations and the moderate-level solution proposed

Despite the disparities identified in this article, there is room to narrow the gaps and create an ideal telehealth landscape for Southeast Asia through ASEAN. The first recommendation is to standardize the regulatory framework. This policy considers that the vast differences in national laws prevent ASEAN from adopting a transnational telehealth scheme. It is therefore recommended that ASEAN member states share digital health transformation blueprints among themselves, which can be coordinated first in ASEAN forums involving the Ministries of Health of ASEAN member states and ASEAN Plus forums (‘ASEAN Plus Three’, for example, would include China, South Korea, and Japan). By synergizing efforts through the agreement over general sets of standards, countries can establish long-term plans to develop relevant government institutions and telehealth programs that would advance the readiness of ASEAN member states in post-pandemic times, albeit in a way that acknowledges the distinct elements of ASEAN’s telehealth environment from those of ASEAN’s partnering states.

A standardized regulatory framework would also establish equal grounds for cybersecurity measures. Since telehealth is a substantial technological development, cybersecurity threats are inevitable. This first recommendation should address ASEAN-identified threats and possible scenarios, and foster cybersecurity frameworks, such as a single certification approach for the Southeast Asian region. Doing so ensures that both ASEAN member states and the private sectors can build synergy in their programs, as slightly mentioned in the ASEAN Digital Masterplan 2025. ^16^ As seen in past reports and studies, telehealth experience in the European Union shows that unified responses can drive significant health trends in the region. ^19, 20^

The second recommendation is for ASEAN to enhance investments in the digital health sector. Given that most digital health initiatives during COVID-19 originated in the private sector, it would be ideal for ASEAN to establish a private-sector-supported telehealth sandbox to work on specific telehealth elements across the region. Collaborating with ASEAN member state governments, donors, and the private sector provides greater exposure to the importance of digital health initiatives in Southeast Asia and enables the exploration of new technological innovations, thereby helping close the digital gap in the long run. Since Indonesia’s ASEAN chairmanship in 2023, ASEAN has placed great emphasis on the ASEAN Indo-Pacific Forum (AIPF) as a platform for government and private sectors to attract foreign investments from ASEAN (and partnering states). ^21– 24^ Platforms such as the AIPF can be fully utilized to address the lack of readily available finances to explore telehealth opportunities within the region.

Investments in the digital health sector should also consider the issue of digital disparity across ASEAN member states. There is therefore a need to address more grassroots concerns about Southeast Asian people’s digital readiness through the provision of high-speed, accessible internet, digital health literacy training and education, knowledge transfer, and capacity-building measures for user-friendly applications.

Recommendations one and two offer a number of positive benefits for ASEAN and its member states. Most importantly, the recommendation to standardize the regulatory framework and facilitate investments in the digital health sector is consistent with the ‘ASEAN Way.’ The ASEAN Way is ASEAN’s foundational norms for the conduct of international relations in Southeast Asia, placing heavy emphasis on non-interference, consensus decision-making, and the pacific settlement of disputes. ^25– 27^ Telehealth is a sector that has been shown to be vital for the Southeast Asian region and will continue to be in a post-pandemic health landscape. The recommended provisions ensure that they do not impose predetermined policies on ASEAN member states in the digital health sector, as they are mainly normative. It merely continues existing efforts to explore other means to accelerate the telehealth landscape in Southeast Asia through ASEAN.

Nevertheless, the proposed recommendations could have some adverse outcomes. For the first recommendation, there should be an expectation of differing commitments, as reflected in the need to regulate the digital health initiative in Southeast Asia. Some ASEAN member states have a strong prospect of emphasizing traditional interventions in medical care ^28^; meanwhile, others may not have the financial capacity to commit to a single framework. The regional organization’s development gap and disparity among ASEAN members are recurring themes. ^29– 31^ The term’ CMLV’ is used to represent the fragility of the newer members of ASEAN, Cambodia, Myanmar, Laos, and Vietnam, abiding by ASEAN’s fast-paced norm development. ^30^ A look at the current state of ASEAN states’ ICT development and telehealth ( Tables 1 and 2) also shows that the CMLV is among the states that struggle to demonstrate readiness to advance their digital health sectors (minus Vietnam, which has accelerated its ICT and economic developments in the past decade). ^32, 33^ The disparity of digital readiness within a state’s borders should also be considered. As Das and Zhang mentioned at the start of the COVID-19 pandemic, those not digitally versed (within a state) have struggled with the vast digital responses imposed by their governments. ^34^

Another concern that equally affects recommendations one and two is the implications of the increased surveillance tools in telehealth. A critical feature of Southeast Asia is the diversity of government systems among member states, which leads to varied human rights practices. If investments are directed to programs that advance application surveillance tools in digital check-in systems and contact-tracing technologies, there would be concerns about privacy infringements. ASEAN is known to be inconsistent with its human rights standards, seeing the heavy emphasis placed on the promotion of human rights (rather than protection) in the ASEAN Intergovernmental Commission on Human Rights, criticisms over the culture and sovereignty-subjected human rights outlined in the ASEAN Human Rights Declaration, and the blind-eye placed in human rights atrocities taking place in Myanmar. ^35, 36^ Nevertheless, concerns such as those in the realms of surveillance and privacy can be countered through the adoption of a regional data privacy charter for health technologies. The second recommendation aims to accelerate the development of the telehealth landscape in Southeast Asian states. Given the sensitivity of human rights in the more authoritarian ASEAN states, the intention to advance digital health could severely backfire on the organization if such technologies are not balanced with proper care for privacy rights.

Given the positive and negative implications of the two recommendations, this article suggests implementing moderate changes to the Southeast Asian telehealth landscape. The impact of digital initiatives on maintaining order and assisting policymakers in responding to pandemics has been undeniable. Nevertheless, Southeast Asia is a unique region. First, Southeast Asia’s demography shows a significant disparity in digital readiness (both government and society). Second, Southeast Asia’s sensitivity to human rights discourses underscores the importance of avoiding overly intrusive policies that could undermine human rights conditions within a state’s borders. The best measure, therefore, is to moderately adopt the first recommendation by the ASEAN standardizing regulatory framework at a pace acceptable to all members. Second, investments in the telehealth sector should ensure that policies include explicit provisions to address the potential misuse of technology (especially in ASEAN member states with authoritarian settings).

## 4. Conclusion

The digital technology landscape of Southeast Asia is rapidly growing. Lessons from the COVID-19 pandemic showed that Southeast Asia is among the regions that place great emphasis on the nexus between technology and health care, aiming to deliver better nationwide health services efficiently. Nevertheless, a significant disparity was identified among Southeast Asian states in digital readiness and the pace of digital health initiatives. As Southeast Asia’s leading regional group, ASEAN has the potential to address lingering concerns that have impeded the region’s acceleration of telehealth to new heights.

This article recommends measures that ASEAN could adopt. First, ASEAN’s regional telehealth framework is standardized by sharing digital health transformation blueprints and by utilizing ASEAN and ASEAN Plus forums to bridge diverse understandings and advance the region’s digital health initiatives. Second, ASEAN is to facilitate investments in the digital health sector, primarily through a telehealth sandbox, and to facilitate finance collaborations across sectors. Nevertheless, it is also pivotal to balance the various pros and cons of the policies. Although both recommendations are consistent with the ASEAN Way, there are still lingering concerns about differing commitment expectations among ASEAN member states, varying capacities of the CMLV, and the risks of privacy infringements and the misuse of technology by authoritarian states in the region. It is therefore recommended that a moderate pace be adopted for telehealth development in Southeast Asia. Despite the positive prospects, ensuring that the recommendations align with Southeast Asia’s unique political landscape should be ASEAN’s top priority.

## Ethical approval

Ethical approval is no required for this article.

## Data Availability

The dataset used in this study is publicly available and sourced from reputable organizations. All data can be accessed through their official platforms, with the detail data and links accessible below:
• CISCO Digital Readiness Index: https://www.cisco.com/c/m/en_us/about/corporate-social-responsibility/research-resources/digital-readiness-index.html#/; • Global Digital Health Monitor: https://digitalhealthmonitor.org/stateofdigitalhealth23 All data required to replicate the findings of this study are available in those websites, in which users can filter based on the inquired variable and period.
