# Supplementary material for: Digital Technologies in a Post-Pandemic Southeast Asia: Measures for Enhancing Regional Approaches
Source: F1000Res. 2025 Dec 1;14:623. Originally published 2025 Jun 25. [Version 2] doi: 10.12688/f1000research.165762.2 (PMC12728336; doi:10.12688/f1000research.165762.2)
Supplement: Peer review history

# Reviewer report: Reviewer response for version 2

**DOI:** 10.5256/f1000research.191869.r437730

**Published:** 2025-12-26

**Authors:** Yuni Intarti

**Affiliations:** 1 Universitas Indonesia, Depok, West Java, Indonesia

I, as a reviewer, have approved the article revisions made by the authors.

Does the paper provide a comprehensive overview of the policy and the context of its implementation in a way which is accessible to a general reader?

Partly

Is the discussion on the implications clearly and accurately presented and does it cite the current literature?

Partly

Are the recommendations made clear, balanced, and justified on the basis of the presented arguments?

Partly

Reviewer Expertise:

regionalism, ASEAN

I confirm that I have read this submission and believe that I have an appropriate level of expertise to confirm that it is of an acceptable scientific standard.

# Reviewer report: Reviewer response for version 2

**DOI:** 10.5256/f1000research.191869.r437732

**Published:** 2025-12-23

**Authors:** Quoc Dung Mai

**Affiliations:** 1 Ho Chi Minh City University of Industry and Trade, Ho Chi Minh City, Vietnam

I agree with the author's revised article, I have no further comments.

Does the paper provide a comprehensive overview of the policy and the context of its implementation in a way which is accessible to a general reader?

Partly

Is the discussion on the implications clearly and accurately presented and does it cite the current literature?

Partly

Are the recommendations made clear, balanced, and justified on the basis of the presented arguments?

Partly

Reviewer Expertise:

History, International Relations, Political Science

I confirm that I have read this submission and believe that I have an appropriate level of expertise to confirm that it is of an acceptable scientific standard.

# Reviewer report: Reviewer response for version 1

**DOI:** 10.5256/f1000research.182503.r428127

**Published:** 2025-11-22

**Authors:** Quoc Dung Mai

**Affiliations:** 1 Ho Chi Minh City University of Industry and Trade, Ho Chi Minh City, Vietnam

- The paper addresses a timely and practically meaningful topic: how ASEAN can strengthen regional cooperation to advance digital health development in the post-pandemic context. The content demonstrates the author’s understanding of ASEAN’s regional mechanisms and the relationship between digital technologies, healthcare transformation, and institutional cooperation. However, the paper remains largely descriptive and lacks deeper analysis of policy coordination and institutional mechanisms among member states.

 - The introduction presents relevant background information and concrete data on ASEAN during the pandemic, allowing readers to understand differences across countries. However, the author does not sufficiently explain the underlying causes of these disparities, such as variations in governance capacity, infrastructure investment, or workforce skills. For international readers, frequent use of internal ASEAN terminology (such as ACRF, AIPF, ASEAN Plus) may be difficult to follow. Including brief explanations of these frameworks would improve the paper’s accessibility.

 - The paper cites credible sources such as ASEAN, WHO, and Cisco, but most data used stops at 2021–2022, whereas digital transformation has advanced rapidly in countries such as Indonesia, Vietnam, and the Philippines. This makes the picture of digital readiness somewhat outdated. The discussion on privacy and surveillance risks is a strength, yet the link between digital readiness and policy implementation remains general and does not clearly identify causes such as fiscal constraints or institutional capacity gaps. The author should consider updating the data (e.g., ICT Development Index 2023–2024 or the World Bank Digital Adoption Index) or explicitly acknowledge the limitations of using earlier datasets.

 - The two key recommendations standardizing regional regulatory frameworks and promoting investment through sandboxes and public private partnerships are reasonable and align with ASEAN’s consensus-based principles. However, the proposals remain broad and lack detail regarding implementation mechanisms, roles of different country groups, and evaluation metrics. The paper should further analyze how lower-capacity members (CMLV countries) can participate and propose indicators such as digital health access rates or the number of cross-border cooperation initiatives.

 - A notable limitation is the lack of analysis on how ASEAN actually coordinates digital health implementation. The paper treats ASEAN as a unified bloc, without clarifying internal coordination mechanisms or the leadership roles of more advanced members (such as Singapore and Thailand). Moreover, key external partners such as the WHO, the World Bank, ADB, and major technology firms are not discussed, despite their growing importance in supporting regional digital capacity.

 - Tác giả nên đưa vào một tiểu mục ngắn về các cơ chế điều phối của ASEAN (ví dụ: Cụm Y tế ASEAN, ASCC, AIPF, ASEAN Plus) và sự tương tác của các cơ chế này với các tổ chức quốc tế. Việc bổ sung một ví dụ so sánh, chẳng hạn như nền tảng eSanjeevani của Ấn Độ, sẽ nâng cao góc nhìn liên khu vực và chiều sâu phân tích.

 - Nhìn chung, bài viết đóng góp giá trị cho việc nghiên cứu quản trị y tế số tại ASEAN. Để tăng cường giá trị học thuật và thực tiễn, tác giả nên cập nhật dữ liệu, phân tích sâu hơn về sự phối hợp giữa các thể chế và đưa ra các khuyến nghị chính sách cụ thể hơn, từ đó nắm bắt tốt hơn tiềm năng hợp tác khu vực trong kỷ nguyên y tế số hậu đại dịch.

Does the paper provide a comprehensive overview of the policy and the context of its implementation in a way which is accessible to a general reader?

Partly

Is the discussion on the implications clearly and accurately presented and does it cite the current literature?

Partly

Are the recommendations made clear, balanced, and justified on the basis of the presented arguments?

Partly

Reviewer Expertise:

History, International Relations, Political Science

I confirm that I have read this submission and believe that I have an appropriate level of expertise to confirm that it is of an acceptable scientific standard, however I have significant reservations, as outlined above.

# Response

**Published:** 2025-11-22

**Authors:** bama andika putra

**Affiliations:** University of Bristol School of Sociology Politics and International Studies, Bristol, England, UK

**Comment 1:** The introduction presents relevant background information and concrete data on ASEAN during the pandemic, allowing readers to understand differences across countries. However, the author does not sufficiently explain the underlying causes of these disparities, such as variations in governance capacity, infrastructure investment, or workforce skills. For international readers, frequent use of internal ASEAN terminology (such as ACRF, AIPF, ASEAN Plus) may be difficult to follow. Including brief explanations of these frameworks would improve the paper’s accessibility.

**Response:**  As this is a policy brief, there is not much space to go on the specifics of the causes to issues related to the empirical inquiry. Therefore, a minor revision made is in the last paragraph of section 2, which outlines several of the basic causes of the disparities that focuses on telehealth. The revised paragraph in version 2 of the article: “Digitalization efforts are not evenly distributed across Southeast Asia. The lack of capacity disparity and technological readiness severely limits the ability to respond to future public health crises of a similar magnitude to COVID-19. The region hosts high-tech states that are digital pioneers in telehealth, such as Singapore, but also countries with some of the lowest digital literacy and penetration, such as Cambodia, Myanmar, and Lao PDR. In general, issues of digital readiness related to data governance, funding inequities, and lack of ICT infrastructure remain the lingering challenges for Southeast Asian states. The following section will explore some recommendations that ASEAN could undertake as a regional grouping and provide the suggested actionable recommendations.”

**Comment 2: **The paper cites credible sources such as ASEAN, WHO, and Cisco, but most data used stops at 2021–2022, whereas digital transformation has advanced rapidly in countries such as Indonesia, Vietnam, and the Philippines. This makes the picture of digital readiness somewhat outdated. The discussion on privacy and surveillance risks is a strength, yet the link between digital readiness and policy implementation remains general and does not clearly identify causes such as fiscal constraints or institutional capacity gaps. The author should consider updating the data (e.g., ICT Development Index 2023–2024 or the World Bank Digital Adoption Index) or explicitly acknowledge the limitations of using earlier datasets.

**Response: **In the second article revision, I have decided to update several of the data used. For example, in Table 1, I used the ITU ICT Development Index of 2024, which compares the figures universal connectivity and indicators of the enablers of connectivity. In Table 2, I updated the data to the Global Digital Health Monitor 2023. Several of the revised paragraphs explaining these points: “However, Southeast Asia is a profoundly diverse region. A look into the International Telecommunication Union’s (ITU) 2024 Information, Technology, and Communication (ICT) Development Index (Table 1 below) reveals how the region’s nations have diverse conditions based on the pillars of universal connectivity (indicators related to the people, households, communities, and business) and indicators of the enablers of connectivity (infrastructure, affordability, safety and security, device, and skills) [10]. The comparison below compares the scores between 2023 and 2024 to provide an overview of changes and trends in the region”.

 “Digital solutions have been a prominent feature in Southeast Asia’s health landscape. Collectively, ASEAN and its member states learned from COVID-19 and established the ASEAN BioDiaspora Virtual Center to enhance the regional organization’s response and preparedness for future epidemics and pandemics. ^15 ^As slightly shown in the previous section, however, on an individual scale, Southeast Asian states have taken different approaches to their telehealth. They fulfill the functions of communication, aid distribution, digital contract tracing, and symptom self-reporting related to pandemics and other health concerns. ^1 ^The current state of the ASEAN member states can be seen in the Global Development Incubator’s Global Digital Health Monitor, 2023 (Table 2 below). Southeast Asia’s digital health landscape is so diverse that some states are in Phase 1 (no coordinating body for digital health care/ nascent governance structure) and others in Phase 5 (digital health and data governance institutionalized) [17].”

**Comment 3:** The two key recommendations standardizing regional regulatory frameworks and promoting investment through sandboxes and public private partnerships are reasonable and align with ASEAN’s consensus-based principles. However, the proposals remain broad and lack detail regarding implementation mechanisms, roles of different country groups, and evaluation metrics. The paper should further analyze how lower-capacity members (CMLV countries) can participate and propose indicators such as digital health access rates or the number of cross-border cooperation initiatives.

**Response:** The second article version covers several additional details on the two recommendations. Several explores how similar policies have been adopted in other regions (Example: European Union), clarifying where Vietnam stands in the issue as it technically is distanced with the other MLV states, as well as some potential outcomes to dig deeper on the arguments. The revisions are located in section 3 of the study:

 “Despite the disparities identified in this article, there is room to narrow the gaps and create an ideal telehealth landscape for Southeast Asia through ASEAN. The first recommendation is to standardize the regulatory framework. This policy considers that the vast differences in national laws prevent ASEAN from adopting a transnational telehealth scheme. It is therefore recommended that ASEAN member states share digital health transformation blueprints among themselves, which can be coordinated first in ASEAN forums involving the Ministries of Health of ASEAN member states and ASEAN Plus forums (‘ASEAN Plus Three’, for example, would include China, South Korea, and Japan). By synergizing efforts through the agreement over general sets of standards, countries can establish long-term plans to develop relevant government institutions and telehealth programs that would advance the readiness of ASEAN member states in post-pandemic times, albeit in a way that acknowledges the distinct elements of ASEAN’s telehealth environment from those of ASEAN’s partnering states.

 A standardized regulatory framework would also establish equal grounds for cybersecurity measures. Since telehealth is a substantial technological development, cybersecurity threats are inevitable. This first recommendation should address ASEAN-identified threats and possible scenarios, and foster cybersecurity frameworks, such as a single certification approach for the Southeast Asian region. Doing so ensures that both ASEAN member states and the private sectors can build synergy in their programs, as slightly mentioned in the ASEAN Digital Masterplan 2025 [16]. As seen in past reports and studies, telehealth experience in the European Union shows that unified responses can drive significant health trends in the region [22,23].

 The second recommendation is for ASEAN to enhance investments in the digital health sector. Given that most digital health initiatives during COVID-19 originated in the private sector, it would be ideal for ASEAN to establish a private-sector-supported telehealth sandbox to work on specific telehealth elements across the region. Collaborating with ASEAN member state governments, donors, and the private sector provides greater exposure to the importance of digital health initiatives in Southeast Asia and enables the exploration of new technological innovations, thereby helping close the digital gap in the long run. Since Indonesia’s ASEAN chairmanship in 2023, ASEAN has placed great emphasis on the ASEAN Indo-Pacific Forum (AIPF) as a platform for government and private sectors to attract foreign investments from ASEAN (and partnering states) [24–26]. Platforms such as the AIPF can be fully utilized to address the lack of readily available finances to explore telehealth opportunities within the region.”

 “Nevertheless, the proposed recommendations could have some adverse outcomes. For the first recommendation, there should be an expectation of differing commitments, as reflected in the need to regulate the digital health initiative in Southeast Asia. Some ASEAN member states have a strong prospect of emphasizing traditional interventions in medical care [30]; meanwhile, others may not have the financial capacity to commit to a single framework. The regional organization's development gap and disparity among ASEAN members are recurring themes [31–33]. The term' CMLV' is used to represent the fragility of the newer members of ASEAN, Cambodia, Myanmar, Laos, and Vietnam, abiding by ASEAN’s fast-paced norm development [32]. A look at the current state of ASEAN states’ ICT development and telehealth (Tables 1 and 2) also shows that the CMLV is among the states that struggle to demonstrate readiness to advance their digital health sectors (minus Vietnam, which has accelerated its ICT and economic developments in the past decade) [20,21]. The disparity of digital readiness within a state’s borders should also be considered. As Das and Zhang mentioned at the start of the COVID-19 pandemic, those not digitally versed (within a state) have struggled with the vast digital responses imposed by their governments [34].”

**Comment 4: **A notable limitation is the lack of analysis on how ASEAN actually coordinates digital health implementation. The paper treats ASEAN as a unified bloc, without clarifying internal coordination mechanisms or the leadership roles of more advanced members (such as Singapore and Thailand). Moreover, key external partners such as the WHO, the World Bank, ADB, and major technology firms are not discussed, despite their growing importance in supporting regional digital capacity.

**Response:** The argument proposed is mainly normative. Meaning, any elements towards technical coordination mechanisms would be a follow up study, as the intention of this research is to cover the importance of unified policies amid potential outbreaks in the future. In regards to external partners, due to issues of word limitation (as it is a policy brief), I limit the discussions of external partners mentioned by the reviewer intentionally, as its inclusion would warrant a deeper analysis as to each stakeholder (WHO, WB, ADB, etc.)

# Reviewer report: Reviewer response for version 1

**DOI:** 10.5256/f1000research.182503.r420713

**Published:** 2025-11-12

**Authors:** Yuni Intarti

**Affiliations:** 1 Universitas Indonesia, Depok, West Java, Indonesia

Bama Andika Putra’s article reviews how the ASEAN nations have used telehealth to respond to COVID-19 and suggests regional policy solutions to address gaps in readiness for the digital age. The author finds that while COVID-19 has catalyzed telehealth across Southeast Asia with some success, it has also exposed deep disparities in the region and within the group of ASEAN nations which must be bridged going forward if telehealth is to remain sustainable in the long run. The article recommends exactly how to sustain the ASEAN processes while bridging those gaps. The author suggests that two types of major regional policy offer have the potential to close the gap: first, the ASEAN framework must be used to build telehealth as a shared responsibility within and beyond the organization by sharing national digital health blueprints through ASEAN facilitation and joint dialogue between ASEAN and “ASEAN Plus” members to align cyber law and standards, cybersecurity. Similarly, ASEAN aims to encourage more telehealth investments and innovation, for example by establishing a regional telehealth sandbox that provides positive regulatory and financing conditions for new technologies to be piloted in public-private partnerships. The author stresses multiple times that all of this activity is still within the “ASEAN Way,” and no deviations from the member state are pushed. In the conclusions, the author openly admits that they even suggest if it would make sense to hold back commitments based on impactful capacity and willingness levels, or to worry about privacy or possible authoritarian system misuse, but then re-frames this situation in the conclusions. In the implications and recommendations, the author ultimately recommends a moderate approach to applying the framework among all states at their own pace, with privacy safeguards if referred to: the balance among urgent digital health implementation and the narrowing access to significantly for digital measures should remain the same. The article concludes with the recommendation that regional policy must be careful and slow because Southeast Asia’s digital technology capacity and governance understanding is so diverse.

 1. Comprehensiveness and accessibility of the policy context: partly. The priorities for enhancing the policy overview’s completeness and currency for general readers include: a) Update or contextualize data on digital readiness. Provide new indicators from more recent sources or explain that the Cisco 2021 figures are a pre-pandemic baseline. This will get rid of “static” as a part of the title and make it seem less like development, while the reader will easily understand the state of much of the ASEAN data this article uses. b) Broaden the comparative context. Relate to known and loved telehealth successes when discussing ASEAN’s efforts. c) Clarify policy background details. Explain what that is, in lay terms, CMLV minority representative in ASEAN—as the article does on numerous occasions but is unsure if every term has been covered. Be very clear how the ASEAN Digital Masterplan is distinct from ASEAN Plus Three. This will maintain the accessibility of the article while adding more depth to its policy content.

 2. Clarity of implications and use of current literature: partly. The paper does discuss the implications of its proposals, and it does use a wide swathe of literature. However, the proposals could be more clearly supported by up-to-date sources. It adds a section that examines the implications of the recommendations, which is open on both sides. For example, some member states may not have the means or the institutions to implement common standards, and for that matter, not every country wants every aspect of their health run by the internet; even Malaysia lacks the broadband capacity to support telehealth.

 Finally, the number of sources used in the paper is appropriate, with over 30 references to other academic sources, World Bank reports, and other authoritative documents. However, the author could aim to find slightly more up-to-date literature that bears obvious relevance to the implications of the problem. While many sources are used from 2020-2021, it is a justifiable focus due to the digital health issues arising in the early times of the pandemic. Regarding the discussion clarity, the author successfully expresses key points. However, at times, they may seem a little too scattered; for example, concepts of “ different commitments ” and a source on the ASEAN development gap are referred to several times.

 Ways to improve the quality: a) Develop analytical explanations in greater detail: Include more specific implications of the disparities discovered regarding the telehealth policy. For example, how a low ICT infrastructure or poor governance in one of the states would prevent the successful implementation of a telehealth program over the region; can be supported with a recent case study or analysis. b) Use more up-to-date sources: If possible, use some recent evaluations to prove impact and current achievements, post-COVID digital health progress reports, or academic research on the telemedicine adoption. c) Offer policies and studies proving the potential for improvement: Include the examples of implementation where negative implications were rapidly reduced. For example, where all the surveillance worries exist, the author can point to examples of regional policy papers or critiques suggesting the implementation of data privacy standards. d) Work on eliminating redundancy: To avoid repeating the same ideas, combine the information on the ASEAN Way benefits and commitment of the member states, and active participation of the human rights experts, etc.

 Clarity, balance, and justification of recommendations: Partly. The article’s two main policy recommendations are clearly and derived from the problems identified above and the author does attempt to balance them with cautionary notes. However, the recommendations section is only partly satisfactory: although the article justifies the recommendations, some elements of justification are missing or could be expanded. A stronger justification could refer to existing evidence of how standardization has affected other regions or other sectors if any studies exist (e.g. the European Union’s experience of e-health and interoperability). Similarly, while recommendation 2 is justified by the need for investment, it introduces the concept of a “telehealth sandbox,” which is not explained above or supported by any evidence of its successful implementation in ASEAN. Additionally, the financial underpinning of a “telehealth sandbox” remains unmentioned – would it rely on donor funding, and how would benefits be shared with countries of more questionable digital ecosystem strength? Due to the absence of these two elements, the recommendations feel only somewhat justified – theoretically sound but without enough evidence to believe that they can be carried out successfully.

 Recommendations for improvement:

 a) Strengthen the evidence base for each recommendation: A little more explanation or an example or two to show why these interventions are likely to work is needed (Recommendation 1 and 2).

 b) Address feasibility and implementation steps: Acknowledge the implementation challenges and suggest a way to overcome them. For instance, suggest a phased approach for the regulatory framework: first, agree on a general set of standards, then offer countries support in building their capacity to comply. Explain how the sandbox would be funded: could some of the funds come from ASEAN’s existing development programs or from international donors partnered with ASEAN? Finally, discuss which ASEAN body will coordinate these efforts: will it be a new ASEAN working group, or will the ASEAN Health Ministers Meeting be charged with coordinating?

 c) Center equity and inclusion: Rather than fully abstract the recommendations immediately above, the author could include provisions within their design requiring them to assist weaker member states more explicitly. For instance, in the case of Recommendation 2, the mandate of the telehealth investment initiative could specify the closing of the digital gap – or connectivity to non-capital cities – including rural and under-served communities and digital literacy programs in Cambodia, Laos, Myanmar, etc. Integrate these protections into the recommendation plan: to fully justify the recommendations given the possibility of failure, the author should add policy safeguards. For example, Recommendation 1 could add the development of a region-wide data privacy standard or even more guards, like interoperability guidelines that restrict sharing of data per privacy safeguards. Similarly, Recommendation 2 could add governance for the sandbox – does ASEAN have its digital health ethics board?

 d) Edit for length and repetition: While reading through the annotated draft, the author can look for passages that only summarise without also justifying the point. Several sentences in this section may rehash variations of the measure-justify-next step structure; for example, some sentences in this section seemed solely to paraphrase the introduction/Section 2. Each recommendation’s justification should be succinct and focused on explication and the next level.

 Key Issues to address for an acceptable Scientific Standard: The author must attend to several concerns raised above to reach an acceptable scholarly standard. Incorporate updated data and sources: using 2021 data without explaining that it is missing 2022 context is a significant limitation. Expand analytic depth: the article’s current version pays lip service to the problem—uneven readiness—and does not explain how and why this manifests. Assess feasibility of recommendations: any but the most cursory commentary should at least consider the feasibility of its proposals. In this case, the author needs at least a token analysis of the political and financial feasibility for his recommendations—for example, how it might be possible to achieve consensus on a framework or how the less developed members can be anticipated to afford and/or adopt their preferred measures. Without feasible reasoning, the recommendations come across as speculative and divorced from practical considerations, which weakens their persuasive power. Address privacy and rights safeguards. Due to valid concerns about surveillance and privacy violations, this might be an opportunity to discuss necessary safeguards. Eliminate redundancy and improve clarity. There are standards for scholarly prose, and one of them is that writing should be concise and to the point. That applies to the recommendations section.

 Strengthening the article through the revisions above is necessary. Only then will this work be sufficiently comprehensive, sufficiently grounded in current conditions to make effective recommendations, and robustly persuadent. Without the necessary background and contextual awareness, this works fall far short of the level of scientific rigor to be maximally helpful to the ASEAN telehealth discourse. The author can improve the comprehensibility and utility of the manuscript by enhancing the data support, analytical depth, and practical recommendations contained within the text so that decision-makers and readers can benefit from the knowledge provided.

 Note:

 (Be careful about including Vietnam in the group of underdeveloped (less developed) ASEAN countries (CLMV), based on OECD's data, Vietnam has had the fastest economic growth among ASEAN countries over the past five years and already possesses a high level of IT technology. Given Vietnam's economic and technological growth, I believe it's time to move Vietnam out of the CLMV group).

 Wishing you all the best.

Does the paper provide a comprehensive overview of the policy and the context of its implementation in a way which is accessible to a general reader?

Partly

Is the discussion on the implications clearly and accurately presented and does it cite the current literature?

Partly

Are the recommendations made clear, balanced, and justified on the basis of the presented arguments?

Partly

Reviewer Expertise:

regionalism, ASEAN

I confirm that I have read this submission and believe that I have an appropriate level of expertise to confirm that it is of an acceptable scientific standard, however I have significant reservations, as outlined above.

# Response

**Published:** 2025-11-19

**Authors:** bama andika putra

**Affiliations:** University of Bristol School of Sociology Politics and International Studies, Bristol, England, UK

Thank you for the comments. Please find below my responses:

**Reviewer 2, comment 1 (a)** : Update or contextualize data on digital readiness. Provide new indicators from more recent sources or explain that the Cisco 2021 figures are a pre-pandemic baseline. This will get rid of “static” as a part of the title and make it seem less like development, while the reader will easily understand the state of much of the ASEAN data this article uses.

**Response: **I agree with this suggestion. CISCO no longer updates their figures, as in 2023-2024, they have changed to cover a more specific index on AI readiness, rather than digital readiness. Consequently, several of the previous figures that this article benefits from using, no longer can be used due to the different components assessed. I have decided to use the ITU’s ICT development index of 2024, which provides an overview on ICT usage (prospects and challenges), and allows comparison between Southeast Asian states. The revised paragraph in (third paragraph of section 1): “However, Southeast Asia is a profoundly diverse region. A look into the International Telecommunication Union’s (ITU) 2024 Information, Technology, and Communication (ICT) Development Index (Table 1 below) reveals how the region’s nations have diverse conditions based on the pillars of universal connectivity (indicators related to the people, households, communities, and business) and indicators of the enablers of connectivity (infrastructure, affordability, safety and security, device, and skills) [10]. The comparison below compares the scores between 2023 and 2024 to provide an overview of changes and trends in the region”

**Reviewer 2, comment 1(b):** Broaden the comparative context. Relate to known and loved telehealth successes when discussing ASEAN’s efforts

**Response: **In the second last paragraph of the introduction section, I outline several examples of the telehealth platforms established within the region, which has notably attracted positive responses by the community. The explanation covering this: “A contributor to such disparities is the ASEAN member states' undertaking of telehealth initiatives at their own pace and initiative. Take, for example, health consultation applications such as Halodoc (Indonesia), elderly health monitoring such as DoCare Protect (Thailand), coordination of community support for dementia patients like Dementia Friends application (Singapore), and the diagnosing of patients through chat such as OneNUHS Health Chatbox (Singapore) and CekMata (Indonesia) [11–15]. During the COVID-19 pandemic, Indonesia’s PeduliLindungi and Malaysia’s Mysejahtera were influential in providing individual COVID-19-related information to all its citizens [16].” Furthermore, as means to display more ‘Asian’ and universal trend of telehealth, I have incorporated a notable example from India’s success in ‘eSanjevaani’.

**Reviewer 2, comment 1(c):** Clarify policy background details. Explain what that is, in lay terms, CMLV minority representative in ASEAN—as the article does on numerous occasions but is unsure if every term has been covered. Be very clear how the ASEAN Digital Masterplan is distinct from ASEAN Plus Three. This will maintain the accessibility of the article while adding more depth to its policy content.

**Reviewer 2, comment 2:** The paper does discuss the implications of its proposals, and it does use a wide swathe of literature. However, the proposals could be more clearly supported by up-to-date sources. It adds a section that examines the implications of the recommendations, which is open on both sides. For example, some member states may not have the means or the institutions to implement common standards, and for that matter, not every country wants every aspect of their health run by the internet; even Malaysia lacks the broadband capacity to support telehealth.

 Finally, the number of sources used in the paper is appropriate, with over 30 references to other academic sources, World Bank reports, and other authoritative documents. However, the author could aim to find slightly more up-to-date literature that bears obvious relevance to the implications of the problem. While many sources are used from 2020-2021, it is a justifiable focus due to the digital health issues arising in the early times of the pandemic. Regarding the discussion clarity, the author successfully expresses key points. However, at times, they may seem a little too scattered; for example, concepts of “different commitments” and a source on the ASEAN development gap are referred to several times.

**Response: **On the first point, I would argue that the reviewer’s case on Malaysia is in contrast to the result of my research. A look into several recently published literatures shows that Malaysia is more than capable in enhancing their telehealth environment and are not impeded by broadband capacity. Examples: https://www.sciencedirect.com/science/article/pii/S3050837125000311

https://www.healthcareitnews.com/news/asia/2000-telehealth-kiosks-be-deployed-rural-malaysia

 Therefore, although this is a high possibility case in rural areas of Malaysia, the evidence does not show an obvious pattern of concern, which therefore leads me to exclude this example.

 On the second point, yes, I have updated the two tables used for this study. To replace CISCO’s digital readiness index 2021, I opted for the ITU ICT development index 2024. Meanwhile, Table 2’s figure is updated to the 2023 figures (unfortunately, no published figure for 2024 is currently available).

 On the third point, the structure follows the journal’s policy brief recommendation, and starts by exploring the policy options, why they are selected, and the positive and negative implications. This is followed by an explanation on which solution is preferred in the study. Therefore, although the idea of commitment is scattered, it still follows the structure intended.

**Reviewer 2, comment 3:**

 a) Develop analytical explanations in greater detail: Include more specific implications of the disparities discovered regarding the telehealth policy. For example, how a low ICT infrastructure or poor governance in one of the states would prevent the successful implementation of a telehealth program over the region; can be supported with a recent case study or analysis.

 b) Use more up-to-date sources: If possible, use some recent evaluations to prove impact and current achievements, post-COVID digital health progress reports, or academic research on the telemedicine adoption.

 c) Offer policies and studies proving the potential for improvement: Include the examples of implementation where negative implications were rapidly reduced. For example, where all the surveillance worries exist, the author can point to examples of regional policy papers or critiques suggesting the implementation of data privacy standards.

 d) Work on eliminating redundancy: To avoid repeating the same ideas, combine the information on the ASEAN Way benefits and commitment of the member states, and active participation of the human rights experts, etc.

**Response: **In the revised fifth paragraph of section 3, I explore how the differences of taken policies is a possibility, however, as the context is ASEAN, there is an expectation that the introduced policies are normative in nature, rather than imposing. The revised paragraph: “Recommendations one and two offer a number of positive benefits for ASEAN and its member states. Most importantly, the recommendation to standardize the regulatory framework and facilitate investments in the digital health sector is consistent with the ‘ASEAN Way.’ The ASEAN Way is ASEAN’s foundational norms for the conduct of international relations in the Southeast Asian region, placing heavy emphasis on non-interference, consensus decision-making, and the pacific settlement of disputes [27–29]. Telehealth is a sector that has been shown to be vital for the Southeast Asian region and will continue to be in a post-pandemic health landscape. The recommended provisions ensure that it does not impose policies that would pressure ASEAN member states to adopt predetermined policies in the digital health sector, as the provisions themselves are mainly normative. It merely continues existing efforts to explore other means to accelerate the telehealth landscape in Southeast Asia through ASEAN.”

 On the second point, the data has been updated to the 2023 and 2024 figures. On the third point, paragraphs 2 and 3 explores these ideas, by looking at recent reports and publications that cover ASEAN’s existing regulatory frameworks in telehealth, and the potential relevance of the AIPF to be utilized on this occasion

**Reviewer 2, comment 4(a)** : However, the recommendations section is only partly satisfactory: although the article justifies the recommendations, some elements of justification are missing or could be expanded. A stronger justification could refer to existing evidence of how standardization has affected other regions or other sectors if any studies exist (e.g. the European Union’s experience of e-health and interoperability).

**Reviewer 2, comment 5(a):** Strengthen the evidence base for each recommendation: A little more explanation or an example or two to show why these interventions are likely to work is needed (Recommendation 1 and 2).

**Response: **I have added several recent reports and studies suggesting how the EU’s telehealth environment increased due to introduced regional initiatives (revisions in the second paragraph of section 3). The revisions: “A standardized regulatory framework would also establish equal grounds for cybersecurity measures. Since telehealth is a substantial technological development, cybersecurity threats are inevitable. This first recommendation should address such ASEAN-identified threats and possible scenarios, fostering cybersecurity frameworks such as a single certification approach for the Southeast Asian region. Doing so ensures that both ASEAN member states and the private sectors can build synergy in their programs, as slightly mentioned in the ASEAN Digital Masterplan 2025 [16]. As seen in past reports and studies, the experience of telehealth in the European Union shows that unified responses can lead to significant health trends in the region [22,23].”

**Reviewer 2, comment 4(b)** : Similarly, while recommendation 2 is justified by the need for investment, it introduces the concept of a “telehealth sandbox,” which is not explained above or supported by any evidence of its successful implementation in ASEAN. Additionally, the financial underpinning of a “telehealth sandbox” remains unmentioned – would it rely on donor funding, and how would benefits be shared with countries of more questionable digital ecosystem strength? Due to the absence of these two elements, the recommendations feel only somewhat justified – theoretically sound but without enough evidence to believe that they can be carried out successfully.

**Reviewer 2, comment 4(c)** : Center equity and inclusion: Rather than fully abstract the recommendations immediately above, the author could include provisions within their design requiring them to assist weaker member states more explicitly. For instance, in the case of Recommendation 2, the mandate of the telehealth investment initiative could specify the closing of the digital gap – or connectivity to non-capital cities – including rural and under-served communities and digital literacy programs in Cambodia, Laos, Myanmar, etc. Integrate these protections into the recommendation plan: to fully justify the recommendations given the possibility of failure, the author should add policy safeguards. For example, Recommendation 1 could add the development of a region-wide data privacy standard or even more guards, like interoperability guidelines that restrict sharing of data per privacy safeguards. Similarly, Recommendation 2 could add governance for the sandbox – does ASEAN have its digital health ethics board?

**Response:** The second recommendation is not intentionally made to be imposing a particular system or mechanism. The reason for this is that, if for example, the system of financial underpinning is determined for the sandbox, there is the fear that some ASEAN nations would not agree with the specified terms. As seen in Table 1, the disparity of digital readiness, and the disparity of telehealth among ASEAN member states leads to the idea that these states have different national interests as they are in distinct stages of ICT development.

 On the second point of revision, I have revised this paragraph to also mention that the second recommendation can also lead to the closing of the digital gap in the long run. Revised sentences: “The second recommendation is for ASEAN to enhance investments in the digital health sector. Given that most digital health initiatives during COVID-19 originated in the private sector, it would be ideal for ASEAN to establish a private-sector-supported telehealth sandbox to work on specific telehealth elements across the region. Collaborating with ASEAN member state governments, donors, and the private sector provides greater exposure to the importance of digital health initiatives in Southeast Asia and enables the exploration of new technological innovations, which allows the closure of the digital gap in the long run”

**Reviewer 2, comment 5(c)** : Center equity and inclusion: Rather than fully abstract the recommendations immediately above, the author could include provisions within their design requiring them to assist weaker member states more explicitly. For instance, in the case of Recommendation 2, the mandate of the telehealth investment initiative could specify the closing of the digital gap – or connectivity to non-capital cities – including rural and under-served communities and digital literacy programs in Cambodia, Laos, Myanmar, etc. Integrate these protections into the recommendation plan: to fully justify the recommendations given the possibility of failure, the author should add policy safeguards. For example, Recommendation 1 could add the development of a region-wide data privacy standard or even more guards, like interoperability guidelines that restrict sharing of data per privacy safeguards. Similarly, Recommendation 2 could add governance for the sandbox – does ASEAN have its digital health ethics board?

**Response: **I agree with this suggestion. The revisions made is in the second last paragraph of section 3, which explains that concerns on surveillance and privacy can potentially be countered by developing a regional data privacy charter for health technologies. The revised paragraph: “Another concern that equally affects recommendations one and two is the implications of the increased surveillance tools in telehealth. A critical feature of the Southeast Asian region is the diversity of government systems among the member states, leading to diverse human rights appreciation. If investments are directed to programs that advance application surveillance tools in digital check-in systems and contact tracing technologies, there would be a concern about privacy infringements. ASEAN is known to be inconsistent with its human rights standards, seeing the heavy emphasis placed on the promotion of human rights (rather than protection) in the ASEAN Intergovernmental Commission on Human Rights, criticisms over the culture and sovereignty-subjected human rights outlined in the ASEAN Human Rights Declaration, and the blind-eye placed in human rights atrocities taking place in Myanmar [35–38]. Nevertheless, concerns such as that in the realms of surveillance and privacy can be countered trough the adoption of a regional data privacy charter for health technologies. The second recommendation aims to accelerate the telehealth landscape of Southeast Asian states. Considering the sensitivity of human rights in the more authoritarian states of ASEAN, the intention to advance digital health could severely backlash the organization if such technologies are not balanced with proper care over privacy rights.”

**Reviewer 2, comment 6:** Key Issues to address for an acceptable Scientific Standard: The author must attend to several concerns raised above to reach an acceptable scholarly standard. Incorporate updated data and sources: using 2021 data without explaining that it is missing 2022 context is a significant limitation. Expand analytic depth: the article’s current version pays lip service to the problem—uneven readiness—and does not explain how and why this manifests. Assess feasibility of recommendations: any but the most cursory commentary should at least consider the feasibility of its proposals. In this case, the author needs at least a token analysis of the political and financial feasibility for his recommendations—for example, how it might be possible to achieve consensus on a framework or how the less developed members can be anticipated to afford and/or adopt their preferred measures. Without feasible reasoning, the recommendations come across as speculative and divorced from practical considerations, which weakens their persuasive power. Address privacy and rights safeguards. Due to valid concerns about surveillance and privacy violations, this might be an opportunity to discuss necessary safeguards. Eliminate redundancy and improve clarity. There are standards for scholarly prose, and one of them is that writing should be concise and to the point. That applies to the recommendations section.

**Response: **These points are responded in comment specific responses of number 1-5

**Reviewer 2, comment 7:** Be careful about including Vietnam in the group of underdeveloped (less developed) ASEAN countries (CLMV), based on OECD's data, Vietnam has had the fastest economic growth among ASEAN countries over the past five years and already possesses a high level of IT technology. Given Vietnam's economic and technological growth, I believe it's time to move Vietnam out of the CLMV group

**Response: **Thank you for this comment. I agree with this and revised the sixth paragraph by explaining that a notable exception in the CMLV is Vietnam, which has shown great progress towards its economy and technological advancements in the past decade. The revised paragraph: “Nevertheless, the proposed recommendations could have some adverse outcomes. For the first recommendation, there should be an expectation of differing commitments, as reflected in the need to regulate the digital health initiative in Southeast Asia. Some ASEAN member states have a strong prospect of emphasizing traditional interventions in medical care [30]; meanwhile, others may not have the financial capacity to commit to a single framework. The regional organization's development gap and disparity among ASEAN members are recurring themes [31–33]. The term' CMLV' is used to represent the fragility of the newer members of ASEAN, Cambodia, Myanmar, Laos, and Vietnam, abiding by ASEAN’s fast-paced norm development [32]. A look at the current state of ASEAN states’ ICT development and telehealth (Tables 1 and 2) also shows that the CMLV is among the states that struggle to demonstrate readiness to advance their digital health sectors (minus Vietnam, which has accelerated its ICT and economic developments in the past decade) [20,21]. The disparity of digital readiness within a state’s borders should also be considered. As Das and Zhang mentioned at the start of the COVID-19 pandemic, those not digitally versed (within a state) have struggled with the vast digital responses imposed by their governments [34].”

# Reviewer report: Reviewer response for version 1

**DOI:** 10.5256/f1000research.182503.r409548

**Published:** 2025-10-09

**Authors:** Vanita Singh

**Affiliations:** 1 Management Development Institute, Gurugram, India

While the policy brief provides a thoughtful overview of ASEAN’s telehealth landscape, there are notable gaps that affect its comprehensiveness and timeliness.

 -First, the omission of India’s eSanjeevani telehealth platform represents a missed opportunity for regional benchmarking. India’s large-scale, government-led teleconsultation model successfully implemented across multiple states offers valuable lessons in scalability, digital inclusion, and public health integration that could inform ASEAN’s regional strategies, especially for lower-income members seeking to expand access.

 Second, the brief’s reliance on the 2021 CISCO Digital Readiness Index raises concerns about the validity of its conclusions. Considerable progress in digital infrastructure, health informatics, and broadband penetration has occurred in many ASEAN countries since then, particularly in Indonesia, Vietnam, and the Philippines. Citing only 2021 data risks portraying a static view of digital capacity and underrepresents recent advancements driven by post-pandemic recovery investments.

 Given that digital readiness evolves rapidly, the brief would benefit from either integrating updated indicators (e.g., 2023–2024 ICT Development Index or World Bank Digital Adoption Index) or explicitly acknowledging the limitations of using older data. Without this, the policy recommendations may appear misaligned with the current realities of ASEAN’s digital transformation and telehealth maturity.

**-**Analytical Depth is missing. The proposed causal linkages may be explored in-depth. The brief could more explicitly link disparities in digital readiness to the specific challenges of implementing telehealth policy (e.g., data governance, funding inequities, or ICT infrastructure).

**-**While the recommendations are sound, more analysis of their political and financial feasibility at the ASEAN level would be useful. For instance, how might resource-limited countries practically adopt common standards?

**-**The discussion on surveillance and privacy is commendable but could benefit from concrete policy safeguards—for instance; Encouraging ASEAN to develop a Regional Data Privacy Charter for health technologies.

 -Section 3 (“The way forward”) could be more concise—some paragraphs repeat earlier points made in Sections 1 and 2.

Does the paper provide a comprehensive overview of the policy and the context of its implementation in a way which is accessible to a general reader?

Partly

Is the discussion on the implications clearly and accurately presented and does it cite the current literature?

Partly

Are the recommendations made clear, balanced, and justified on the basis of the presented arguments?

Partly

Reviewer Expertise:

Digital health; health policy, public health

I confirm that I have read this submission and believe that I have an appropriate level of expertise to confirm that it is of an acceptable scientific standard, however I have significant reservations, as outlined above.

# Response

**Published:** 2025-11-19

**Authors:** bama andika putra

**Affiliations:** University of Bristol School of Sociology Politics and International Studies, Bristol, England, UK

**Reviewer 1, comment 1:**

 First, the omission of India’s eSanjeevani telehealth platform represents a missed opportunity for regional benchmarking. India’s large-scale, government-led teleconsultation model successfully implemented across multiple states offers valuable lessons in scalability, digital inclusion, and public health integration that could inform ASEAN’s regional strategies, especially for lower-income members seeking to expand access.

**Response: **I agree with this comment. I revised the second last paragraph of the introduction section to explain how the vast development of digital health platforms in Southeast Asia follows the success of other platforms in Asia, such as that of eSanjeevani. The revised paragraph: “A contributor to such disparities is the ASEAN member states' undertaking of telehealth initiatives at their own pace and initiative. Take, for example, health consultation applications such as Halodoc (Indonesia), elderly health monitoring such as DoCare Protect (Thailand), coordination of community support for dementia patients like Dementia Friends application (Singapore), and the diagnosing of patients through chat such as OneNUHS Health Chatbox (Singapore) and CekMata (Indonesia) [11–15]. During the COVID-19 pandemic, Indonesia’s PeduliLindungi and Malaysia’s Mysejahtera were influential in providing individual COVID-19-related information to all its citizens [16]. As Clavier and Ghesquiere mentioned, “Digital solutions have been the signature of Southeast Asia’s response to COVID-19” [1]. These initiatives are extensions to already successful telehealth platform’s in Asia such as India’s *eSanjeevani*, which promotes inclusivity through the bridging of the urban-rural divide through equitable healthcare access for all [16]”

**Reviewer 1, comment 2:**

 Second, the brief’s reliance on the 2021 CISCO Digital Readiness Index raises concerns about the validity of its conclusions. Considerable progress in digital infrastructure, health informatics, and broadband penetration has occurred in many ASEAN countries since then, particularly in Indonesia, Vietnam, and the Philippines. Citing only 2021 data risks portraying a static view of digital capacity and underrepresents recent advancements driven by post-pandemic recovery investments. Given that digital readiness evolves rapidly, the brief would benefit from either integrating updated indicators (e.g., 2023–2024 ICT Development Index or World Bank Digital Adoption Index) or explicitly acknowledging the limitations of using older data. Without this, the policy recommendations may appear misaligned with the current realities of ASEAN’s digital transformation and telehealth maturity.

**Response: **I have revised the Table 1 to include the results of the ITU’s ICT Development Index 2024 as means to show how diverse the region’s universal connectivity and enablers of connectivity indicators. It does so by displaying figures between 2023 and 2024 (to show trends), and how Southeast Asian states’ scores differently with one another (to reflect disparities). The changed paragraph explaining this (third paragraph of the introduction section): “However, Southeast Asia is a profoundly diverse region. A look into the International Telecommunication Union’s (ITU) 2024 Information, Technology, and Communication (ICT) Development Index (Table 1 below) reveals how the region’s nations have diverse conditions based on the pillars of universal connectivity (indicators related to the people, households, communities, and business) and indicators of the enablers of connectivity (infrastructure, affordability, safety and security, device, and skills) [10]. The comparison below compares the scores between 2023 and 2024 to provide an overview of changes and trends in the region”

**Reviewer 1, comment 3:**

 Analytical Depth is missing. The proposed causal linkages may be explored in-depth. ****The brief could more explicitly link disparities in digital readiness to the specific challenges of implementing telehealth policy (e.g., data governance, funding inequities, or ICT infrastructure).

**Response: **I agree that linkages needed to be made more in-depth related to the disparities in digital readiness. In the last paragraph of section 2, I revised the arguments to incorporate the high possibility that disparities occurs due to issues of data governance, funding inequities, and lack of ICT infrastructures as lingering challenges for Southeast Asian states.

**Reviewer 1, comment 4** : While the recommendations are sound, more analysis of their political and financial feasibility at the ASEAN level would be useful. For instance, how might resource-limited countries practically adopt common standards?

**Response: **The recommendations 1 and 2 provided, as this is the context of ASEAN, are in the forms of normative suggestions that could be adopted by ASEAN. The revisions made in the fifth paragraph of section 3 emphasizes this, meanwhile, the previous paragraph also acknowledges that if there are financial difficulties encountered, one potential solution is to accelerate collaborations between the different stakeholders within a state. The revised paragraph: “Recommendations one and two offer a number of positive benefits for ASEAN and its member states. Most importantly, the recommendation to standardize the regulatory framework and facilitate investments in the digital health sector is consistent with the ‘ASEAN Way.’ The ASEAN Way is ASEAN’s foundational norms in the conduct of international relations in the Southeast Asian region, by placing heavy emphasis on the importance of non-interference, consensus decision-making, and pacific settlement of disputes [27–29]. Telehealth is a sector that has shown to be vital for the Southeast Asian region and will continue to be in a post-pandemic health landscape. The recommended provisions ensure that it does not impose policies in which ASEAN member states would be pressured to undertake pre-determined policies concerning the digital health sector, as the provisions itself are mainly normative in nature. It merely continues existing efforts to explore other means to accelerate the telehealth landscape in Southeast Asia through ASEAN”

**Reviewer 1, comment 5:** The discussion on surveillance and privacy is commendable but could benefit from concrete policy safeguards—for instance; Encouraging ASEAN to develop a Regional Data Privacy Charter for health technologies.

 Reviewer 2, comment 5(c): Center equity and inclusion: Rather than fully abstract the recommendations immediately above, the author could include provisions within their design requiring them to assist weaker member states more explicitly. For instance, in the case of Recommendation 2, the mandate of the telehealth investment initiative could specify the closing of the digital gap – or connectivity to non-capital cities – including rural and under-served communities and digital literacy programs in Cambodia, Laos, Myanmar, etc. Integrate these protections into the recommendation plan: to fully justify the recommendations given the possibility of failure, the author should add policy safeguards. For example, Recommendation 1 could add the development of a region-wide data privacy standard or even more guards, like interoperability guidelines that restrict sharing of data per privacy safeguards. Similarly, Recommendation 2 could add governance for the sandbox – does ASEAN have its digital health ethics board?

**Response:** I agree with this suggestion. The revisions made is in the second last paragraph of section 3, which explains that concerns on surveillance and privacy can potentially be countered by developing a regional data privacy charter for health technologies. The revised paragraph: “Another concern that equally affects recommendations one and two is the implications of the increased surveillance tools in telehealth. A critical feature of the Southeast Asian region is the diversity of government systems among the member states, leading to diverse human rights appreciation. If investments are directed to programs that advance application surveillance tools in digital check-in systems and contact tracing technologies, there would be a concern about privacy infringements. ASEAN is known to be inconsistent with its human rights standards, seeing the heavy emphasis placed on the promotion of human rights (rather than protection) in the ASEAN Intergovernmental Commission on Human Rights, criticisms over the culture and sovereignty-subjected human rights outlined in the ASEAN Human Rights Declaration, and the blind-eye placed in human rights atrocities taking place in Myanmar [35–38]. Nevertheless, concerns such as that in the realms of surveillance and privacy can be countered trough the adoption of a regional data privacy charter for health technologies. The second recommendation aims to accelerate the telehealth landscape of Southeast Asian states. Considering the sensitivity of human rights in the more authoritarian states of ASEAN, the intention to advance digital health could severely backlash the organization if such technologies are not balanced with proper care over privacy rights.”

**Reviewer 1, comment 6:**

 Section 3 (“The way forward”) could be more concise—some paragraphs repeat earlier points made in Sections 1 and 2.

**Response: **Revised throughout the section.
